# Evaluation of esterase and hemolysin activities of different *Candida* species isolated from vulvovaginitis cases in Lorestan Province, Iran

**DOI:** 10.29252/cmm.3.4.1

**Published:** 2017-12

**Authors:** Maryam Noori, Mohammad Dakhili, Asghar Sepahvand, Nader Davari

**Affiliations:** 1 Faculty of Medicine, Qom Branch, Islamic Azad University, Qom, Iran; 2 Razi Herbal Medicines Research Center, Lorestan University of Medical Sciences, Khorramabad, Iran; 3 Department of Hematology, Ahvaz Jundishapur University of Medical Sciences, Ahvaz, Iran

**Keywords:** *Candida albicans*, Esterase, Hemolysin, Vulvovaginal candidiasis, Virulence factor

## Abstract

**Background and Purpose:**

Annually affecting millions of women, vulvovaginal candidiasis (VVC) is commonly described by signs and symptoms of vulvovaginal inflammation in the presence of *Candida* species. Today, the detection of the virulence factors plays a major role in the understanding of pathogenesis of candidiasis and helps produce new anticandidial drugs to improve its treatment efficiency. Herein, we aimed to evaluate the esterase and hemolysin activities of the vaginal isolates of *Candida* and their relationship with the presence of VVC.

**Materials and Methods:**

One-hundred vaginal clinical specimens were randomly collected during September-December 2016. The target population consisted of married women suspected of VVC who presented to health centers in Lorestan Province, Iran. In this study, the esterase activity and hemolysin production of* Candida* clinical isolates were evaluated using the Tween 80 opacity test and the plate assay, respectively.

**Results:**

The most frequent *Candida* species was *C. albicans* (66; 66%), followed by *C. glabrata* (11; 11%) and *C. tropicalis* (11; 11%). The highest esterase activity was found in *C. krusei* (75%), followed by *C. albicans* (68.2%) and *C. glabrata* (54.5%). The greater part of the positive esterase isolates had Pz 4+ scores. Among the *Candida* species,* C. albicans (22.7*%*), C. glabrata* (63.6%), and* C. krusei *(50%) were found to have the highest rates of alpha, beta, and gamma hemolysin production, respectively. The level of hemolytic activity in 51% of the *Candida* species was Pz 4+ scores.

**Conclusion:**

According to our results, the higher expression rates of both enzymes in *C. albicans* species relative to those of non-albicans *Candidate* species can partly reflect the role of the virulence factors involved in *C. albicans* pathogenicity.

## Introduction

Vaginal candidiasis (genital/vulvovaginal candidiasis [VVC]) also termed as vaginal yeast infection is commonly known as the second leading cause of vaginitis after bacterial vaginosis [1-3]. While* C. albicans* is considered the most frequent species causing VVC, other *Candida* species are the etiologic agents of half of other fungal infections [4, 5]. 

Reviews have reported that some virulence factors such as germ tube formation, adhesins, phenotypic switching, biofilm formation, and the synthesis of hydrolytic enzymes can contribute to candidiasis [6, 7]. On the other hand, occurrence of these virulence factors can differ depending on the type of species, environmental source, the site and phase of infection, as well as host response to the infection [8]. 

Currently, the identification of virulence factors may play a key role in determining the pathogenesis of candidiasis and introducing new anticandidial agents to promote treatment strategies [8]. Reviews have recounted that* Candida *spp. can secrete a number of exoenzymes for example phospholipase, esterase, hemolysin, and proteinase that are required to colonize and attack host organs [9-11]. 

In Iran, Pakshir et al. (2013) examined 84 *Candida* isolates from onychomycosis and oral lichen planus patients, the majority of which had diverse enzymatic patterns, and *C. parapsilosis* strains had less phospholipase activity [12].

In the current study, we attempted to evaluate the esterase and hemolysin activities of vaginal isolates of *Candida* collected during September-December 2016. The target population consisted of married women suspected of VVC who visited health centers in Lorestan Province, Iran. Further, we investigated the relationship between esterase and hemolysin activities of the vaginal isolates and the presence of VVC.

## Materials and Methods

***Clinical isolates***

One-hundred vaginal clinical specimens were randomly collected during September-December 2016 from married women suspected of VVC who presented to health centers in Lorestan Province, Iran. According to the Centers for Disease Control and Prevention (CDC) guidelines, the indicative factors for the laboratory and clinical diagnosis of VVC include (i) a wet slide or gram stain of vaginal discharge and (ii) a culture or other examination resulting in yeast species identification. Vaginal sampling of the participants was carried out by using a sterile swab using the trained researcher. The isolates were cultured immediately on to Sabouraud Dextrose Agar (SDA). The standard strains of *C. albicans* (Persian Type Culture Collection, PTCC 5027) and *C. glabrata* (CBS 138) were gifted from Department of Medical Mycology, Iran University of Medical Sciences, Tehran, Iran. 

***Candida identification***

In this study, the detection of *Candida* clinical isolates was approved by the conventional mycological techniques, namely the germ tube test in serum, microscopical morphology, chlamydospore formation in Corn Meal Agar (Oxoid, Basingstoke, UK) supplemented with Tween 80, and assimilation of carbon sources via the commercial kit ID 32C (bioMérieux, France) [13].

***Inoculum preparation***

*Candida* clinical isolates were grown on SDA plates for 24 h at 37°C. Then, suitably grown microorganisms were inoculated in sterile saline (0.85%) and standardized based on turbidity to 5 Â 103 CFU (McFarland no: 0.5) per well in RPMI medium under sterile conditions. Serial dilutions were prepared in 100 µl RPMI medium with an equal amount of test samples, and 100 µl of each of the microorganism suspensions was pipetted into each well and incubated at 37°C for 24 h [14]. 

***Esterase activity***

In this study, the esterase activity of* Candida* clinical isolates was evaluated using the Tween 80 opacity test according to the method described by **Fatahinia et al.** [15]. Initially, 10 g of bacteriological peptone, 5 g of sodium chloride, 0.1 g of calcium chloride, and 15 g of agar were dissolved in 1000 ml of distilled water. The medium was autoclaved and gradually cooled to about 50°C. Then, 5 ml of autoclaved Tween 80 was added to the medium and distributed in 8-cm sterile plates. Again, 10 µl of each *Candida* suspension (10^6^ cells/ml) was spot-inoculated on plates and incubated at 30°C and checked on a daily basis for 10 days. All the inoculations were performed in duplicate. The colony diameter (a) and the diameter of colony plus precipitation zone (b) were recorded to measure esterase activity [16].

***Hemolysin activity***

In order to evaluate the hemolysin production of *Candida* clinical isolates, we used the plate assay based on the method explained by Manns et al. [17]. In short, the *Candida* isolates were cultured on Sabouraud Glucose Agar (Merck, Darmstadt, Germany). Afterwards, a suspension of each isolate was prepared in phosphate buffer solution (PBS) and turbidity was adjusted to 0.5 McFarland standard (10^6^ cells/ml). Then, 10 µl of the suspension spot was inoculated on a 3% sugar-enriched sheep blood agar medium. The media were then incubated at 37°C in 5% CO_2_ for two days. Finally, the greenish black ring around each colony was considered as incomplete (alpha) and ring of lysis was considered as complete (beta); on the other hand, the lack of greenish black ring around each colony was considered as no hemolysin activity (gamma).

***Statistical analysis***

Chi-squared test was used to assess the correlation of enzyme activity with the presence of VVC. The assessment of the normality of data was performed by Explore test. The statistical analyses were carried out in SPSS, version 17. *P-value* less than 0.05 was considered statistically significant. 

***Ethical statement***

The present study was approved by the Ethics Committee of Lorestan University of Medical Sciences. A written informed consent was obtained from all the participants before sampling. 

## Results

One-hundred vaginal clinical specimens were included in the present study; the mean age of the participants was 29±3.1 years (range: 18 to 46 years). Fifty-nine (59%) patients demonstrated clinical manifestations such as itching and pruritus (71%), white discharge (59%), and pain during intercourse (20%) as the most common symptoms. The highest prevalence of *Candida* was observed in women aged between 27 and 35 years (43%). Chi-squared test reflected a significant association between the prevalence of *Candida *species and age (*P=0.01*). Six species including *C. albicans* (66; 66%)*, C. tropicalis* (11; 11%)*, C. glabrata* (11; 11%), *C. krusei *(8; 8%)*, C. parapsilosis *(2; 2%)*, *and *C. lipolytica *(2; 2%) were isolated.

**Figure 1:**
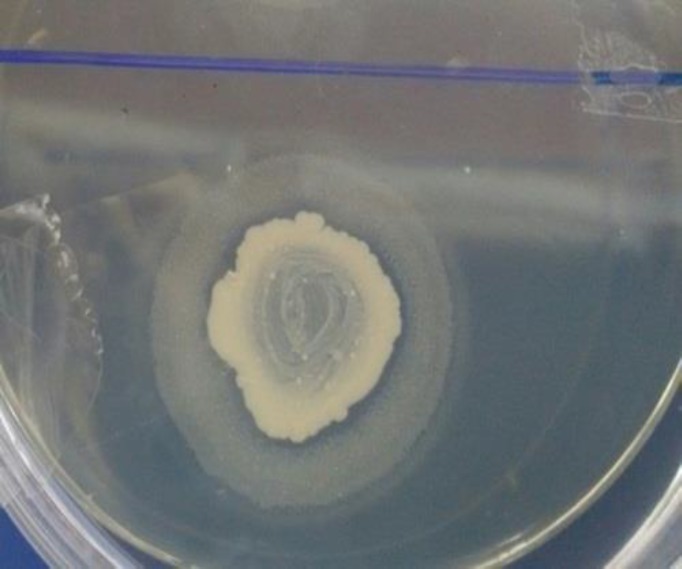
Evaluation of esterase activity and sedimentary zone around *Candida *spp

**Figure 2:**
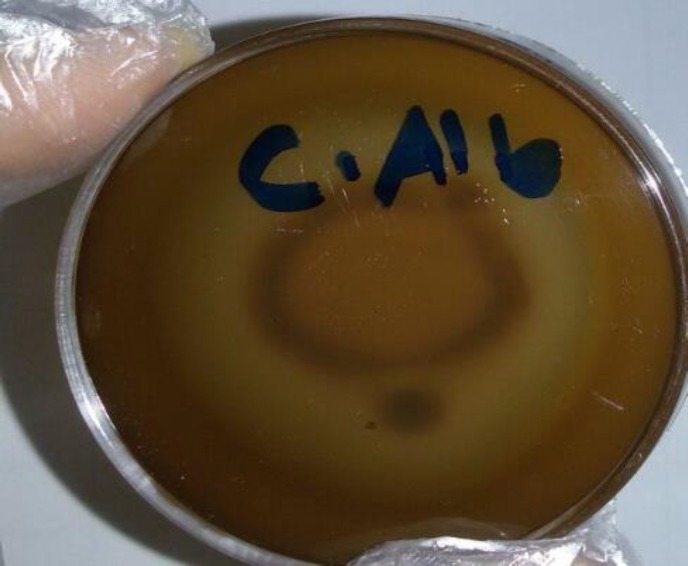
Evaluation of hemolysin activity and sedimentary zone around *Candida albicans*

***Esterase activity***

We evaluated the esterase activity of* Candida* clinical isolates using the Tween 80 opacity test medium (Figure 1). As shown in Table 1, the highest esterase activity was found in *C. krusei* (75%), followed by *C. albicans* (68.2%) and C. *glabrata* (54.5%). The majority of the positive esterase isolates had Pz 4+ scores. Among the *Candida* species, only C. *parapsilosis* did not show any esterase activity using the Tween 80 opacity test medium. The obtained results demonstrated no statistically significant relationship between esterase production by *Candida* species and the presence of VVC (*P=0.44*). Furthermore, esterase activity was Pz 4+ scores in *C. albicans* (PTCC 5012) and *C. glabrata* (CBS 138), which were used as controls.

***Hemolysin activity***

The hemolysin production of *Candida* clinical isolates was assessed by the plate assay (Figure 2). Among the clinical isolates, 19%, 46%, and 35% of the *Candida *isolates were able to produce the alpha, beta, and gamma hemolysins, respectively. As shown in Table 2, among the *Candida* species,* C. albicans (22.7*%*), C. glabrata* (63.6%), and* C. krusei *(50%) were found to have the highest rates of alpha, beta, and gamma hemolysin production, respectively.

The level of hemolytic activity in 51% of the *Candida* species was Pz 4+ scores, while in 4% and 11% of the species the levels of hemolytic activity were Pz 3+ and 2+ scores, respectively. *C. krusei, C. parapsilosis, *and *C. lipolytica* showed hemolytic activity only in Pz 4+ scores (Table 3). The obtained results demonstrated no significant relationship between hemolysin production by *Candida* species and the presence of VVC (P=0.98). Furthermore, hemolysin production was Pz 4+ scores in *C. albicans* (PTCC 5012) and *C. glabrata* (CBS 138), which were used as controls.

**Table 1:** Comparison of esterase enzyme activity in different *Candida* species isolated from vulvovaginal candidiasis

***Candida*** ** spp**	**Esterase activity**			
	**Negative** **No. (%)**	**++** **No. (%)**	**+++** **No. (%)**	**++++** **No. (%)**
*C. albicans*	21 (31.8)	0 (0)	0 (0)	45^*^ (68.2)
*C. glabrata*	5 (45.5)	0 (0)	1 (9.1)	5 (45.5)
*C. tropicalis*	6 (54.5)	0 (0)	0 (0)	5 (45.5)
*C. krusei*	2 (25)	0 (0)	0 (0)	6 (75)
*C. parapsilosis*	2 (100)	0 (0)	0 (0)	0 (0)
C. *lipolytica*	1 (50)	0 (0)	0 (0)	1 (50)
Total	37 (37)	0 (0)	1 (1)	62 (62)

* The difference was statistically significant (*P<0.05*)

**Table 2:** The hemolysin production among the *Candida* clinical isolates

***Candida*** ** spp**	**Hemolysin**		
	**Alpha hemolysin**	**Beta hemolysin**	**Gamma hemolysin**
*C. albicans*	15 (22.7)	28 (42.4)	23^*^ (34.8)
*C. glabrata*	1 (9.1)	7 (63.6)	3 (27.3)
*C. tropicalis*	2 (18.2)	6 (54.5)	3 (27.3)
*C. krusei*	1 (12.5)	3 (37.5)	4 (50)
*C. parapsilosis*	0 (0)	1 (50)	1 (50)
C. *lipolytica*	0 (0)	1 (50)	1 (50)
Total	19 (19)	46 (46)	35 (35)

* The difference was statistically significant (*P<0.05*)

**Table 3:** Comparison of hemolysin activity in different *Candida* species isolated from vulvovaginal candidiasis

***Candida*** ** spp**	**Hemolysin activity**			
	**Negative** **No. (%)**	**++** **No. (%)**	**+++** **No. (%)**	**++++** **No. (%)**
*C. albicans*	22 (33.3)	9 (13.6)	3 (4.5)	32^*^ (48.4)
*C. glabrata*	3 (27.3)	1 (9.1)	1 (9.1)	6 (54.5)
*C. tropicalis*	3 (27.3)	1 (9.1)	0 (0)	7 (63.6)
*C. krusei*	4 (50)	0 (0)	0 (0)	4 (50)
*C. parapsilosis*	1 (50)	0 (0)	0 (0)	1 (50)
C. *lipolytica*	1 (50)	0 (0)	0 (0)	1 (50)
Total	34 (34)	11 (11)	4 (4)	51 (51)

* The difference was statistically significant (*P<0.05*)

## Discussion

VVC, affecting millions of women each year, is commonly known as an infection described by signs and symptoms of vulvovaginal inflammation in the presence of *Candida* species [5]. Today, the detection of virulence factors can play a key role in the understanding of the pathogenesis of candidiasis and help introduce new anticandidial drugs for enhancing therapeutic approaches [7].

Nowadays, it has been proven that over 90% of cases of candidiasis infection are caused by *C. albicans*, *C. glabrata*, *C. parapsilosis, C. tropicalis*, and* C. krusei* [1]. Moreover, previous studies showed that *Candida* isolates have considerably higher extracellular enzyme activities than commensal ones [6]. 

In the present study, the highest rate of infection was observed in the 27-35 age group, which is in agreement with the results of Asadi et al. and Akbarzadeh et al. [18, 19]. The higher level of infection in this age group can be attributed to the higher sexual activity of this age group, the physiological and hormonal changes, and the use of various contraceptive methods.

The obtained findings demonstrated that the most prevalent *Candida* species was *C. albicans* (66; 66%), followed by *C. glabrata *(11; 11%) and *C.* *tropicalis* (11; 11%). Similarly, in several studies in Iran, the USA, and India, it was found that *C. albicans* and *C. glabrata* were the most common *Candida* species among the VVC isolates [20-24]. Our findings were relatively consistent with those of other studies carried out elsewhere, and the few differences observed in the prevalence of various *Candida* species could be due to differences in geographic regions, sexual behaviors, cultures, customs of different nations, as well as differences in the study design, target population, and diagnostic methods.

In this study, we found that 63% of the *Candida* species had esterase activity, whereas the highest esterase activity was found in *C. krusei* (75%), followed by *C. albicans* (68.2%) and C. *glabrata* (50%). In line with our results, Pakshir et al. [12] reported that esterase activity of *C. albicans* and *C. parapsylosis* isolated from onychomycosis and oral lichen planus lesions were 87.5% and 43.7%, respectively. In the study conducted by Kumar et al. [25], esterase activities of *C. albicans, C. tropicalis, C. parapsilosis, C. dublinis*, and *C. lipolytica* isolated from clinical samples were 92.2%, 92.3%, 25.6%, 16.6%, and 100%, respectively. The discrepancy in results could be due to differences in geographic regions, methods of diagnosis, as well as sample size.

Recently, Fattahnia et al. [15] found that the esterase activity of all *Candida* isolates from the oral cavity of diabetic and non-diabetic subjects was 100% in Khuzestan Province, Iran, indicating a higher prevalence rate than our finding. This difference in enzyme activity may be due to the different sites of sample isolation.

In this study, 65% of *Candida* species had hemolytic activity, 19% and 46% of which produced alpha and beta hemolysins, respectively. Among them, the highest production was observed in *C. albicans* with 22.7% and 42.4% for hemolysin alpha and hemolytic beta, respectively. In line with our results, Malcok et al. [26] in Turkey reported that the hemolytic activity levels of *C. albicans*, *C. glabrata*, *C. tropicalis*, *C. parapsilosis*, *C. kiper*, *C. kerosene* and *C. guilliermondii *isolated from clinical specimens were 49.6%, 43.4%, 40.8%, 6.8%, 47.4%, 28.3%, and 21.5%, respectively. Unlike our study, in the study conducted by Manns et al. [17] in India, hemolysin activity in all clinical isolates of *C. albicans, C. tropicalis*, and *C. gillermundi* was 100%. This difference may be due to differences in environmental conditions such as differences in sampling site, geographical area, clinical samples, research method, and host immune status. In our study, hemolysin activity was found in 50% of *C. parapsylosis* isolates, but in other studies, enzyme production was reported negative in this species. Differences in geographical area and target population could be accountable for this discrepancy.

## Conclusion

Although both enzymes were expressed approximately in all* Candida *isolates*, *activity of these enzymes was more remarkable in *C.* *albicans* isolates. The considerable presence of both enzymes in *C. albicans* species relative to non-albicans *Candida* species suggested the role of these factors in the development of diseases caused by these yeast agents; however, further investigations are required to shed light on this mechanism.
